# Lace plant ethylene receptors, AmERS1a and AmERS1c, regulate ethylene-induced programmed cell death during leaf morphogenesis

**DOI:** 10.1007/s11103-015-0356-4

**Published:** 2015-08-19

**Authors:** Gaolathe Rantong, Rodger Evans, Arunika H. L. A. N. Gunawardena

**Affiliations:** Biology Department, Life Sciences Centre, Dalhousie University, 1355 Oxford Street, Halifax, NS B3H 4R2 Canada; Biology Department, Acadia University, 33 Westwood Avenue, Wolfville, NS B4P 2R6 Canada

**Keywords:** *Aponogeton madagascariensis*, Ethylene, Ethylene receptors, Lace plant, Leaf morphology, PCD

## Abstract

The lace plant, *Aponogeton madagascariensis*, is an aquatic monocot that forms perforations in its leaves as part of normal leaf development. Perforation formation occurs through developmentally regulated programmed cell death (PCD). The molecular basis of PCD regulation in the lace plant is unknown, however ethylene has been shown to play a significant role. In this study, we examined the role of ethylene receptors during perforation formation. We isolated three lace plant ethylene receptors AmERS1a, AmERS1b and AmERS1c. Using quantitative PCR, we examined their transcript levels at seven stages of leaf development. Through laser-capture microscopy, transcript levels were also determined in cells undergoing PCD and cells not undergoing PCD (NPCD cells). AmERS1a transcript levels were significantly lower in window stage leaves (in which perforation formation and PCD are occurring) as compared to all other leaf developmental stages. AmERS1a and AmERS1c (the most abundant among the three receptors) had the highest transcript levels in mature stage leaves, where PCD is not occurring. Their transcript levels decreased significantly during senescence-associated PCD. AmERS1c had significantly higher transcript levels in NPCD compared to PCD cells. Despite being significantly low in window stage leaves, AmERS1a transcripts were not differentially expressed between PCD and NPCD cells. The results suggested that ethylene receptors negatively regulate ethylene-controlled PCD in the lace plant. A combination of ethylene and receptor levels determines cell fate during perforation formation and leaf senescence. A new model for ethylene emission and receptor expression during lace plant perforation formation and senescence is proposed.

## Introduction

Programmed cell death (PCD) is a genetically controlled cell suicide that eliminates undesirable cells in most multicellular organisms (Greenberg 1996). PCD occurs throughout normal development in plants; starting from the fertilization of the ovule to death of the whole plant (van Doorn and Woltering 2005), and is involved in processes such as death of the embryonic suspensor (Lombardi et al. 2007), leaf and flower senescence [reviewed by Lim et al. (2007), Rogers (2013)], aerenchyma formation (Gunawardena et al. 2001; Lenochová et al. 2009), tracheary element differentiation (Groover and Jones 1999; Fukuda 2000), dehiscence of anthers (Bonner and Dickinson 1989), root cap shedding (Wang et al. 1996), and perforation formation during leaf morphogenesis in *Monstera* and lace plant (Gunawardena et al. 2004, 2005; Wright et al. 2009; Wertman et al. 2012).

In plants, several genetic components have been associated with PCD: these include receptor-like/Pelle kinases, pattern recognition receptors, stress receptors, reactive oxygen (ROS) sensors, MAPK cascade, hormonal regulators, transcription factors and caspase-like enzymes [reviewed in Rantong and Gunawardena (2015)]. Hormones involved in plant PCD include, but are not limited to salicylic acid (Cao et al. 1994; Mur et al. 2013), jasmonic acid (Mur et al. 2013), and ethylene (Zhao and Schaller 2004; Dauphinee et al. 2012).

The phytohormone ethylene has been implicated as an important regulator of PCD in plants (Zhao and Schaller 2004). Examples of plant PCD that are thought to involve ethylene include, but are not limited to: the hypersensitive response, organ senescence, aerenchyma formation, leaf and petal abscission, endosperm cell death (Young et al. 1997; reviewed in Bleecker and Kende 2000; Trobacher 2009; Rogers 2013) and perforation formation in the lace plant (Dauphinee et al. 2012). Ethylene has been shown to promote the onset of senescence (Zacarias and Reid 1990; Jing et al. 2005) and ethylene-insensitive mutants often display delayed senescence (Grbic and Bleecker 1995; Oh et al. 1997; Jing et al. 2005). Also, tomato plants that had suppressed ethylene production showed delayed leaf senescence (John et al. 1995; Jing et al. 2005). Ethylene biosynthesis and action inhibitors have been shown to stop aerenchyma formation in maize roots subjected to low oxygen conditions [reviewed in Drew et al. (2000)]. Also, low concentrations of ethylene induced PCD in cells pre-determined to die during aerenchyma formation (Drew et al. 2000). These examples demonstrate the importance of ethylene in PCD and the significance of both ethylene and PCD during plant development.

Once produced, ethylene is recognised through a signal transduction pathway to trigger ethylene inducible responses. It is recognised by a five-member family of membrane-bound receptors in *Arabidopsis thaliana* found on the endoplasmic reticulum (ER): ETR1, ETR2, ERS1, ERS2 and EIN4 [reviewed in Chang and Stadler (2001), Wang et al. (2002)]. The ethylene receptors act constitutively to negatively regulate the ethylene signal transduction pathway and suppress ethylene responses; hence, decreasing the number of ethylene receptors increases the cell’s sensitivity to ethylene [reviewed in Trobacher (2009)].

Ethylene receptors are homologous to bacterial two-component histidine kinases, which typically consist of two proteins: a sensor histidine kinase and a response regulator (Wurgler-Murphy and Saito 1997; Pirrung 1999; Wang et al. 2002). In *A. thaliana*, there are five ethylene receptors (Hua et al. 1998; Wang et al. 2002). The *A. thaliana* ethylene receptor family can be divided into two subfamilies: ETR1-like subfamily (type I) and ETR2-like subfamily (type II) based on structural similarities (Schaller and Bleecker 1995; Hall et al. 2000; Wang et al. 2002). Despite the structural differences, each ethylene receptor appears to be involved in signal transduction and also in inhibiting ethylene responses (Chang et al. 1993; Hua et al. 1998; Hua and Meyerowitz 1998; Sakai et al. 1998; Wang et al. 2003; O’Malley et al. 2005). Less is known regarding the specific role of each receptor subtype; however, in general, at least one subfamily I receptor (either ETR1 or ERS1) is necessary for most ethylene responses (Wang et al. 2003).

**Fig. 1:**
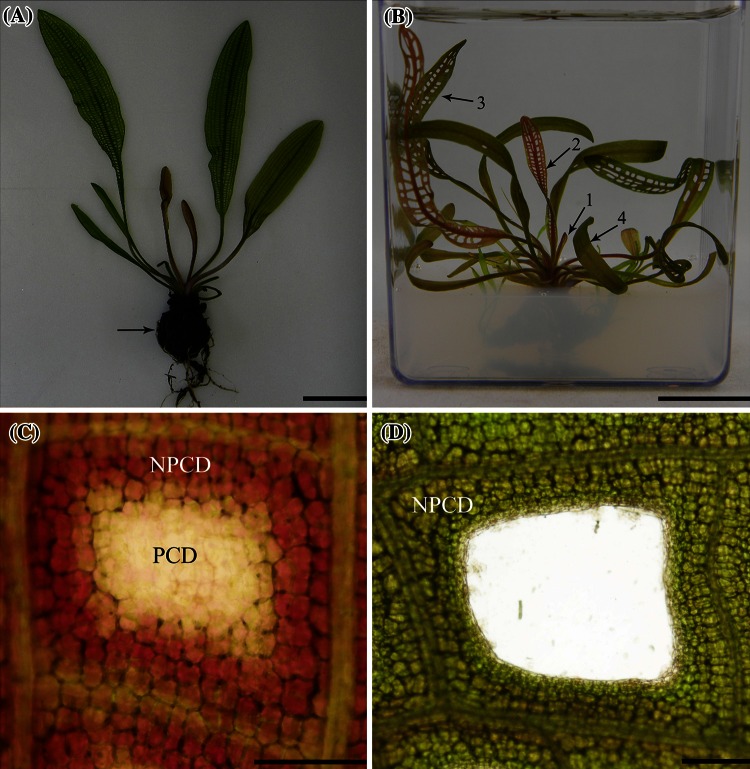
The Lace plant. **a** A typical lace plant from an aquarium. Leaves emerge from a corm (*arrow*). The corm also has several roots which function in anchoring the plant to growth medium. **b** Lace plant growing in a magenta box. This method of growing lace plant was developed to propagate lace plant in axenic conditions. Different developmental stage leaves, such as leaf number 1 (preperforation), 2 (late window) and 3 (mature), as shown in the magenta box grown plant, were harvested and used in experiments. The first few leaves produced by the lace plant do not form perforations (leaf number 4). **c** An areole from a “window” stage leaf, in which perforations are actively forming, depicting 4–5 cell layers of non-dying (NPCD) cells around the perforation site and dying (PCD) cells within the perforation site. **d** A perforation site, with NPCD located between the perforation and vascular tissue. *Bars* 5 cm in **a**, 2.7 cm in **b**, 500 μm in **c** and 150 μm in **d**

The lace plant is a submerged aquatic monocot belonging to the family Aponogetonaceae and employs PCD during leaf morphogenesis (Fig. 1a). The plant forms perforations in its leaves through PCD and can be grown in magenta boxes in axenic conditions for experimental purposes (Gunawardena et al. 2006; Fig. 1b). The formation of perforations in lace plant leaves has been previously characterised and divided into five developmental stages (Gunawardena et al. 2004). In “window” stage leaves, cells at the center of a perforation site (PCD cells; Fig. 1c) begin to undergo PCD. These cells lose their pigmentation and appear somewhat transparent compared to their non-dying (NPCD) counterparts, which turn pink due to high amounts of anthocyanin. The NPCD cells do not undergo PCD during perforation formation and occupy 4–5 cells layers away from vascular tissue (Fig. 1c, d). The process of perforation formation and the morphological aspects of PCD in lace plant have been well studied (Gunawardena et al. 2004, 2005, 2006, 2007; Gunawardena 2008; Wright et al. 2009; Elliott and Gunawardena 2010; Lord et al. 2011; Wertman et al. 2012).

Despite the lace plant being an excellent model for the study of PCD, little to no molecular work has been carried out on the species and the developmental signalling pathways involved during perforation formation remain unclear. However, lace plant leaves undergoing PCD during perforation formation and senescence emit a significantly high amount of ethylene, while inhibition of ethylene biosynthesis aminoethoxyvinylglycine (AVG) inhibits perforation formation in lace plant leaves (Dauphinee et al. 2012). An ethylene receptor inhibitor silver nitrate (AgNO_3_; Gunawardena et al. 2006) was also shown to result in significant reductions in the number of perforations within leaves. These experiments provided indirect evidence for the involvement of ethylene and ethylene receptors in perforation formation. Insight into what signals trigger, and or regulate perforation formation will provide a better understanding of PCD regulation during normal development in plants.

The objective of the following study was to provide more evidence for the involvement of ethylene during lace plant PCD and investigate the role of ethylene receptors in regulation of lace plant PCD. Lace plant ethylene receptors were isolated and their transcript expression patterns were studied in different stages of leaf development and between PCD versus NPCD cells. Based on the results, a model for regulation of PCD during perforation formation and senescence is proposed. This study is the first molecular study of perforation formation via PCD in the lace plant.

## Materials and methods

### Plant materials

Lace plants were propagated under axenic conditions in Magenta GA7 boxes as described by Gunawardena et al. (2006). Plants were grown at 24 °C under daylight simulating fluorescent bulbs (Philips, Daylight Deluxe, F40T12/DX, Markham, Ontario, Canada) providing 12 h light/12 h dark cycles at approximately 125 µmol m^−2^ s^−1^. Leaves at seven different stages of development were selected and harvested from these plants to be used for RNA extraction. For each RNA sample, tissue was collected from at least three leaves obtained from different plants. Analysis was based on data from 28 independent RNA samples (4 RNA samples per leaf developmental stage).

### RNA extraction and cDNA synthesis

The TRI-reagent (Sigma, Oakville, Ontario, Canada) was used for RNA extraction with some modifications to the standard method. Twice the recommended volume of TRI-reagent was used and the RNA pellet was not air-dried. Leaf tissue (without midrib) of approximately 200 mg was used in RNA extraction. The midrib was removed because it contains phenolic compounds, which interfere with RNA extraction. RNA quality for each sample was determined through gel electrophoresis and spectrometry (at 260 nm). RNA was treated with DNase 1 (Fermentas, Burlington, Ontario, Canada) prior to cDNA synthesis, to degrade genomic DNA. cDNA was synthesised using M-MuLV reverse transcriptase (New England Biolabs, Pickering, Ontario, Canada). Two microlitre of RNA, 1 μl of 10 μM dT primer and 1 μl of 10 mM dNTP mix were added to a nuclease free tube. The mixture was then incubated at 65 °C for 5 min in a water bath, quickly chilled on ice and briefly spun to collect the contents. Four microlitre of 5X First Strand Buffer (Invitrogen, Burlington, Ontario, Canada), 1 μl of RNase inhibitor (40 U/μl; New England Biolabs, Pickering, Ontario, Canada) and 2 μl of 0.1 M DTT (Invitrogen, Burlington, Ontario, Canada) were then added to each sample. The mixtures were incubated at 37 °C for 2 min in a water bath. Two microliters of the M-MuLV reverse transcriptase (200 U/μl) was then added and the contents mixed by pipetting. Samples were incubated at 37 °C for 1 h; the reaction was then heat inactivated by incubating the samples at 70 °C for 15 min. Each sample was diluted with nuclease free water to a total volume of 50 μl.

### Laser capture microscopy

In early window and window stage leaves, NPCD cells are pink due to anthocyanin while PCD cells have lost their anthocyanin (Fig. 1c). Therefore, the cell types are easily distinguishable due to their color differences. The cells were separated using a Zeiss PALM Laser Capture Microdissection and Imaging System. A total of 8 different samples (four samples per cell type) were used for RNA extraction, and each sample was collected from at least three different leaves obtained from different plants. RNA was extracted from the cells using a ReliaPrep RNA Cell Miniprep kit (Promega, Nepean, Ontario, Canada), following manufacturer’s instructions. DNase 1 was used to degrade trace amounts DNA, and cDNA was synthesized using Protoscript M-MuLV First Strand cDNA Synthesis Kit (New England Biolabs, Pickering, Ontario, Canada) according to manufacturer’s instructions.

### Isolation of lace plant ethylene receptors

For isolation of lace plant ethylene receptors, cDNA from preperforation, window and mature stage leaves was used. Initial fragments of the ethylene receptors were amplified using forward and reverse degenerate primers; 5′-TGGGTKCTTGTTCAGTTYGGTGC-3′ and 5′-CATTCTCACATGCYTTCCWGTYTC-3′, respectively. These degenerate primers were designed from an alignment of the following sequences; *Arabidopsis thaliana* ecotype Columbia (Col) (NM_105305), *Lycopersicon esculentum* (AF043084), *Oryza sativa* (AB107219), *Pelargonium* × *hortorum* (AF141928), *Vitis vinifera* (AF243474), *Populus trichocarpa* (XM_002302696) and *Physcomitrella patens* ssp. *patens* (XM_001751468). The PCR reaction mixture prepared for amplification consisted of 11.15 μl of nuclease free water, 2 μl 10× Thermobuffer (New England Biolabs, Pickering, Ontario, Canada), 1 μl of 10 mM dNTP mix (New England Biolabs, Pickering, Ontario, Canada), 1 μl of 10 mM forward primer, 1 μl of 10 mM of reverse primer and 0.35 μl of Taq DNA polymerase (5 U/μl) (New England Biolabs, Pickering, Ontario, Canada). As a template, 3.5 μl of cDNA was used. PCR conditions used were 94 °C for 5 min, 40 cycles of 94 °C for 30 s, 45 °C for 30 s and 72 °C for 1 min. Following the 40 cycles, a final primer extension was carried out at 72 °C for 10 min. PCR products were separated on 1.5 % agarose gels containing ethidium bromide (Sigma Aldrich, Oakville, Ontario, Canada) and visualized using DNR F-ChemiBIs 3.2 M Pro (Bio-imaging Systems, Montreal, Quebec, Canada). Amplified products were cloned using the pGEM-T Easy Vector System (Promega, Nepean, Ontario, Canada) following the manufacturer’s instructions. A GenElute plasmid miniprep kit (Sigma, Oakville, Ontario, Canada) was used for plasmid purification. Clones were sent to Macrogen Corp (Rockville, Maryland, USA) for sequencing. The rest of the 3′ end (including 3′ UTR) for each of the ethylene receptors was isolated through 3′-RACE; using an anchored primer (AP; 5′-GGCCACGCGTCGACTAGTACTTTTTTTTTTTTTTTTT-3′) and an abridged universal amplification primer (AUAP; 5′-GTACTAGTCGACGCGTGGCC-3′). An actin gene fragment was also isolated using the degenerate primers 5′-AATGGHACTGGAATGGTCAAGG-3′ and 5′-CAYTTCATGATGGARTTGTA-3′. BioEdit Sequence Alignment Editor (Carlsbad, Ottawa, Ontario, Canada) was used to analyse sequences. Sequences were compared with National Center for Biotechnology Information (NCBI) nonredundant protein (blastx) database sequences for sequence identity analysis.

### Phylogenetic analysis

**Fig. 2:**
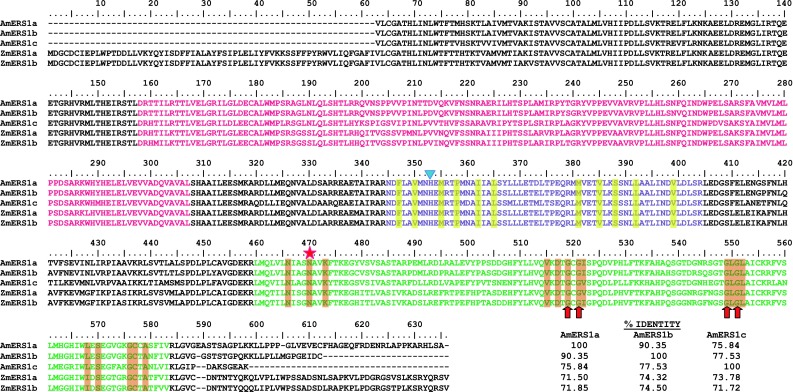
Amino acid sequences of the lace plant ethylene receptors and their alignment to *Z. mays* ERS1a and ERS1b. Several important domains within the ethylene receptors are highlighted; GAF domain (*pink*), histidine kinase domain (*purple*), dimer interface domain (highlighted in *yellow*), HATPase_c (*green*), Mg^2+^ binding site (*red* asteric), G-X-G motif (*red arrows*), phosphorylation site (*blue triangle*), ATP binding site (highlighted in *orange*). The percentage identities of each pair of ethylene receptors are also indicated. Accession numbers: KR349966 (AmERS1a), KR349967 (AmERS1b), KR349968 (AmERS1c), AAR25566 (ZmERS1a) and NP_001137032 (ZmERS1b)

A total of ten ethylene receptor amino acid sequences from maize, rice, and *Arabidopsis* were obtained from the NCBI protein database. GenBank accession numbers of these ethylene receptors are AAR25566 (ZmERS1a), NP_001137032 (ZmERS1b), NP_001104852 (ZmETR2a), XP_008667201 (ZmETR2b), AAB72193 (OsERS1), AAL66363 (OsERS2), CAD39679 (OsETR2), AAL29303 (OsETR3), AAQ07254 (OsETR4) and NP_187108.1 (AtEIN4). These amino acid sequences were aligned to the three lace plant amino acid sequences obtained here using MEGA version 6.06 (Tamura et al. 2007). Prior to phylogenetic tree construction, the large gap at the 5′ end of lace plant sequences (see Fig. 2), and corresponding amino acids in the reference sequences, were deleted. A single tree was constructed, with the *A. thaliana* sequence designated as an outgroup, using the Neighbor-Joining method in MEGA version 6.06. Branch strength within the resulting tree was calculated using 1000 replicates in a nonparametric bootstrap test.

### Quantitative PCR

AmERS1a primers used in QPCR are: 5′-TGATCAGGTAGCAGTTGCTC-3′ and 5′-AGCCTC TCTTCGAGCTGAGTCC-3′. AmERS1c primers used are 5′-AGATCAGGTTGCCGTTGCCC-3′ and 5′-CTAGCTGCATCCAAGGCAAC-3′. 5′-TGATCAGGTAGCTGTTGCAC-3′ and 5′-TGCCTCTCGTCGTGCAGAGTCT-3′ were used for AmERS1b QPCR. For actin QPCR, 5′-TACGACAGGTATCGTGCTTG-3′ and 5′-CAAGCACGATACCTGTCGTA-3′ were used. Prior to QPCR, each primer pair was verified to produce a single amplicon through PCR. The fragments amplified by each of the primer pairs were cloned, sequenced and verified. For QPCR, DNA standards and cDNA samples were amplified using a QuantiTect SYBR Green PCR Kit (Qiagen, Mississauga, Ontario, Canada) following the manufacturer’s instructions. For negative controls, the reverse transcriptase was omitted in the cDNA synthesis reactions and these samples were also subjected to QPCR. Thermal cycling and fluorescence detection were performed using a Rotor-Gene 3000 system (Corbett Research, Sydney, Australia). The QPCR was performed in 20 μl reaction volume and PCR conditions were initial holding at 95 °C for 15 min, 40 cycles of denaturing at 95 °C for 20 s, annealing temperature (59 °C for AmERS1a, 60 °C for AmERS1b, AmERS1c and actin) for 30 s and elongation at 72 °C for 30 s. Melting temperature of the PCR product was monitored after completion of PCR and was used as an indicator that a single specific product was amplified and is responsible for the total fluorescence. The fluorescence was measured at the end of each cycle and standard curves were used to determine mRNA copy numbers of actin and each of the ethylene receptors, as explained in Bustin et al. (2005). Relative steady-state levels of ethylene receptor transcripts were determined by dividing their absolute copy numbers by the copy number of actin transcripts in each sample.

### Statistical analysis

The Quantitative PCR data was analysed via GraphPad Prism version 5.00 (San Diego, CA, USA). The relative abundance of transcripts encoded by each gene is presented as mean ± SEM. A one-way ANOVA was used to determine whether there was a significant difference in relative abundance of transcripts among leaf developmental stages. A Tukey’s HSD test was used to conduct post hoc comparisons. For relative transcript levels between PCD and NPCD cells, an unpaired *t* test was used. Data was determined to be statistically significant if *P* < 0.05.

## Results

### Lace plant ethylene receptors

Three lace plant ethylene receptors were isolated, namely AmERS1a, AmERS1b and AmERS1c. AmERS1a fragment is 1890 bp (including the 3′ untranslated region; KR349966), and translated into a 572 amino acid protein fragment (Fig. 2). AmERS1b fragment was 1867 bp (including the 3′ untranslated region; KR349967), translating into a 549 amino acid fragment (Fig. 2). AmERS1c was 1604 bp (KR349968) and translated into a protein fragment of 534 amino acids. The ethylene receptors shared conserved domains, sites and motifs, such as, the GAF domain, histidine kinase domain, dimer interface domain, HATPase_c, Mg^2+^ binding site, G-X-G motif, phosphorylation site, and adenosine triphosphate (ATP) binding site. These are also conserved in *Z. mays* subfamily I ethylene receptors (Fig. 2). Amongst themselves, lace plant ethylene receptors share high levels of amino acid sequence identity. AmERS1a amino acid fragment shares 90.35 and 75.84 % identities with AmERS1b and AmERS1c respectively. AmERS1b and AmERS1c share 77.53 % identity. The percentage identity between the lace plant and *Z. mays* subfamily I ethylene receptors ranged between 71.5 and 74.5 %.

### Structural features of lace plant ethylene receptors

**Fig. 3:**
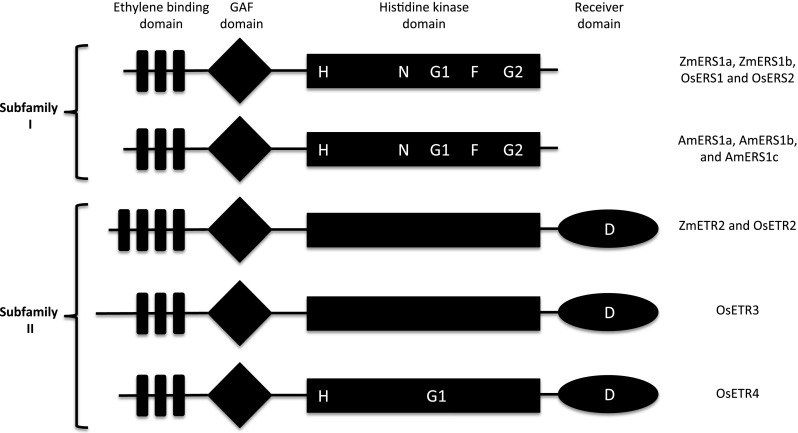
The structure of *Z. mays*, *O. sativa* and lace plant ethylene receptors. ZmERS1a, ZmERS1b, OsERS1 and OsERS2 share a similar structure consisting of an ethylene binding domain, a GAF domain and a functional histidine kinase domain. The lace plant ethylene receptors, AmERS1a, AmERS1b and AmERS1c also share this similar structure. The lace plant ethylene receptors also posses the conserved essential residues (H, N, G1, F and G2), within the histidine kinase domain., ZmETR2b and OsETR2, lack these essential residues, posses an additional hydrophobic transmembrane region within the ethylene-binding domain and has a C-terminal receiver domain. The receiver domain has a conserved phosphorylated aspartate (D) residue. OsETR3 and OsETR4 also have a receiver domain, and lack all essential or some of the essential residues within the histidine kinase domain. ZmERS1a, ZmERS1b, OsERS1 and OsERS2 are subfamily I, while ZmETR2a, ZmETR2b, OsETR2, OsETR3 and OsETR4 are subfamily II ethylene receptors

Lace plant ethylene receptors shared the same structural characteristics with each other (Fig. 3). Compared with rice and maize ethylene receptors, they shared more characteristics with subfamily I (ZmERS1a, ZmERS1b, OsERS1 and OsERS2) than subfamily II receptors (ZmETR2, OsETR2, OsETR3 and OsETR4). They posses the conserved essential residues (H, N, G1, F and G2) within the histidine kinase domain, characteristic of subfamily I receptors, and required for histidine kinase activity. Subfamily II maize and rice receptors lack some or all of the essential residues within the histidine kinase activity. Within all these lace plant ethylene receptors, there is part of the ethylene binding domain, the GAF domain, and a functional histidine kinase domain. They lack a C-terminal receiver domain, which is a response regulator and is present in maize and rice subfamily II ethylene receptors.

**Fig. 4:**
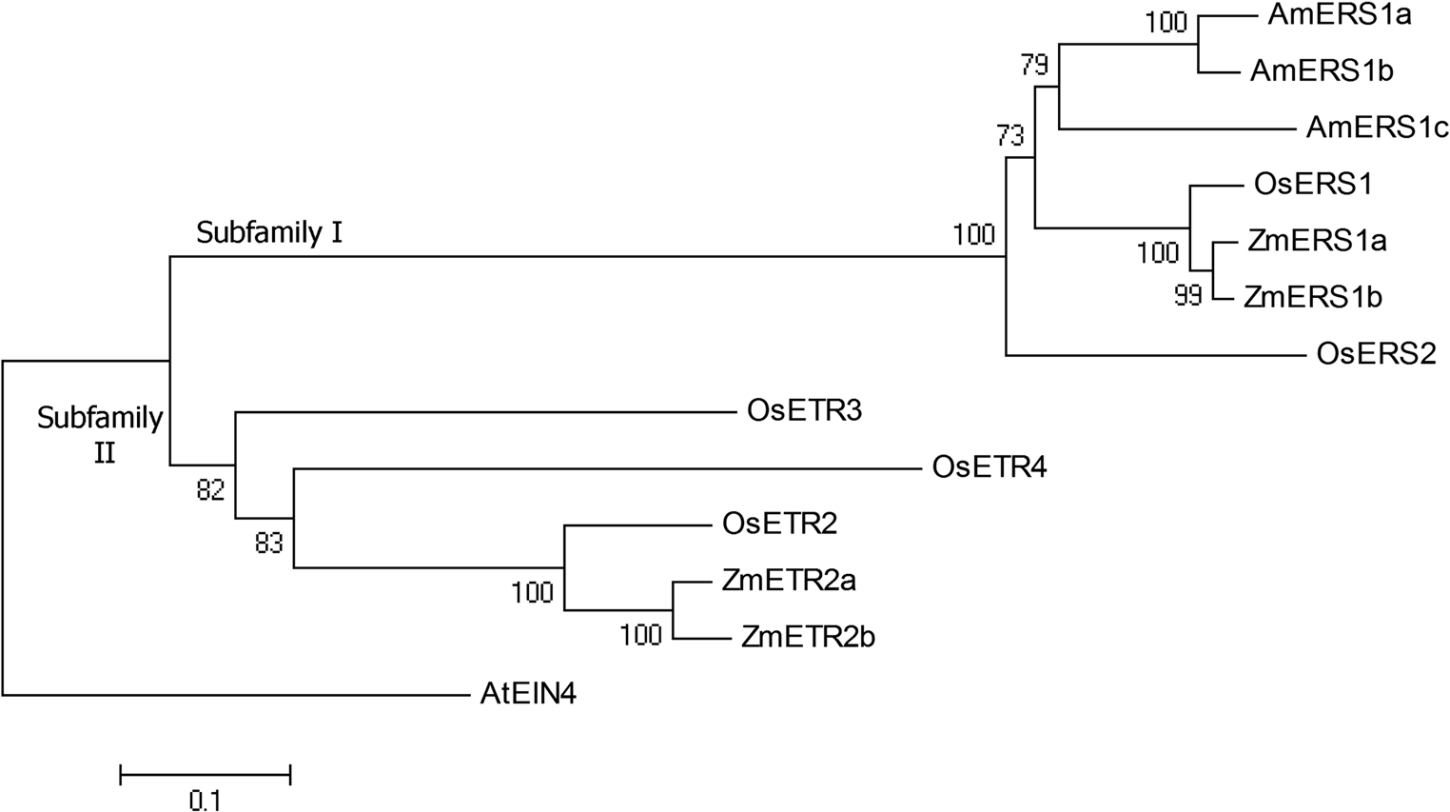
A phylogenetic tree composed of lace plant, rice and maize ethylene receptors. The GenBank accession numbers of the amino acid sequences used are KR349966 (AmERS1a), KR349967 (AmERS1b), KR349968 (AmERS1c), AAR25566 (ZmERS1a), NP_001137032 (ZmERS1b), NP_001104852 (ZmETR2a), XP_008667201 (ZmETR2b), AAB72193 (OsERS1), AAL66363 (OsERS2), CAD39679 (OsETR2), AAL29303 (OsETR3), AAQ07254 (OsETR4) and NP_187108.1 (AtEIN4). *Bar* represents the gap separation distance

A phylogenetic analysis consisting of maize, rice and lace plant ethylene receptors showed that the three lace plant ethylene receptors (AmERS1a, AmERS1b and AmERS1c) are more closely related to each other than they are to other monocot receptors (Fig. 4). AmERS1a and AmERS1b are more closely related to each other than they are to AmERS1c. The three lace plant ethylene receptors are more closely related to subfamily I than subfamily II maize and rice ethylene receptors. Within subfamily I, they are also more closely related to the ERS1 receptors (ZmERS1a, ZmERS1b, OsERS1) than OsERS2.

### AmERS1a, AmERS1b and AmERS1c expression levels in different stages of lace plant leaf development

**Fig. 5:**
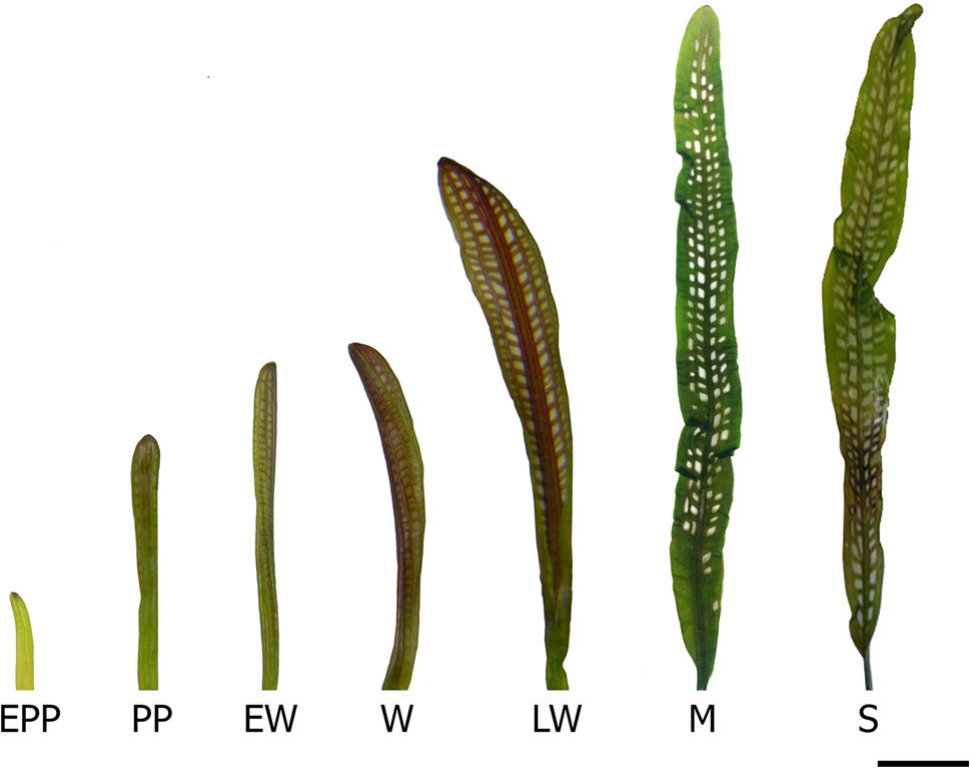
Lace plant leaf development. For experimental purposes, lace plant leaf development was divided into seven stages; early preperforation (EPP), preperforation (PP), early window (EW), window (W), late window (LW), mature (M) and senescence (S). Perforation formation and PCD are occurring during early window, window and late window stages. PCD is also occurring in senescent stage leaves. *Bars* 0.7 cm (EPP-LW) and 1.3 cm (M and S)

To provide insights into the role of ethylene receptors in lace plant leaf development and PCD, quantitative PCR was performed to determine transcript levels of each of the receptors during seven stages of lace plant leaf development (Fig. 5). Stage 1 (early preperforation; EPP), the leaves are young, tightly furled and have just emerged from the corm. There are no visible signs of PCD or perforation formation at this stage. Stage 2 (preperforation; PP), the leaves are still furled, vasculature is well pronounced, but there are still no signs of PCD or perforation formation. During stage 3 (early window; EW), about half of the leaf is unfurled and perforation sites are visible. Cells that do not undergo PCD (NPCD cells) during perforation formation appear pink (due to the pigment anthocyanin) while PCD cells that are destined to die during perforation formation have already lost anthocyanin. In stage 4 (window; W), the entire leaf is unfurled; perforation sites start to become somewhat transparent (PCD cells appear to lose some of their chlorophyll; Fig. 1c). During stage 5 (late window; LW), actual holes start to form at the perforation sites, as some of the cells have died and disintegrate. Some cells at the perforation border are still undergoing PCD. Stage 6 is the mature stage (M), where perforations are fully formed, there are no more signs of PCD and leaves are completely green again. At this stage, only NPCD cells remain, and they occupy 4–5 cell layers between the perforation and vascular tissue (Fig. 1d). The last stage, stage 7 (senescence; S), the leaves are starting to yellow and there are some brown spots on the leaf blade.

**Fig. 6:**
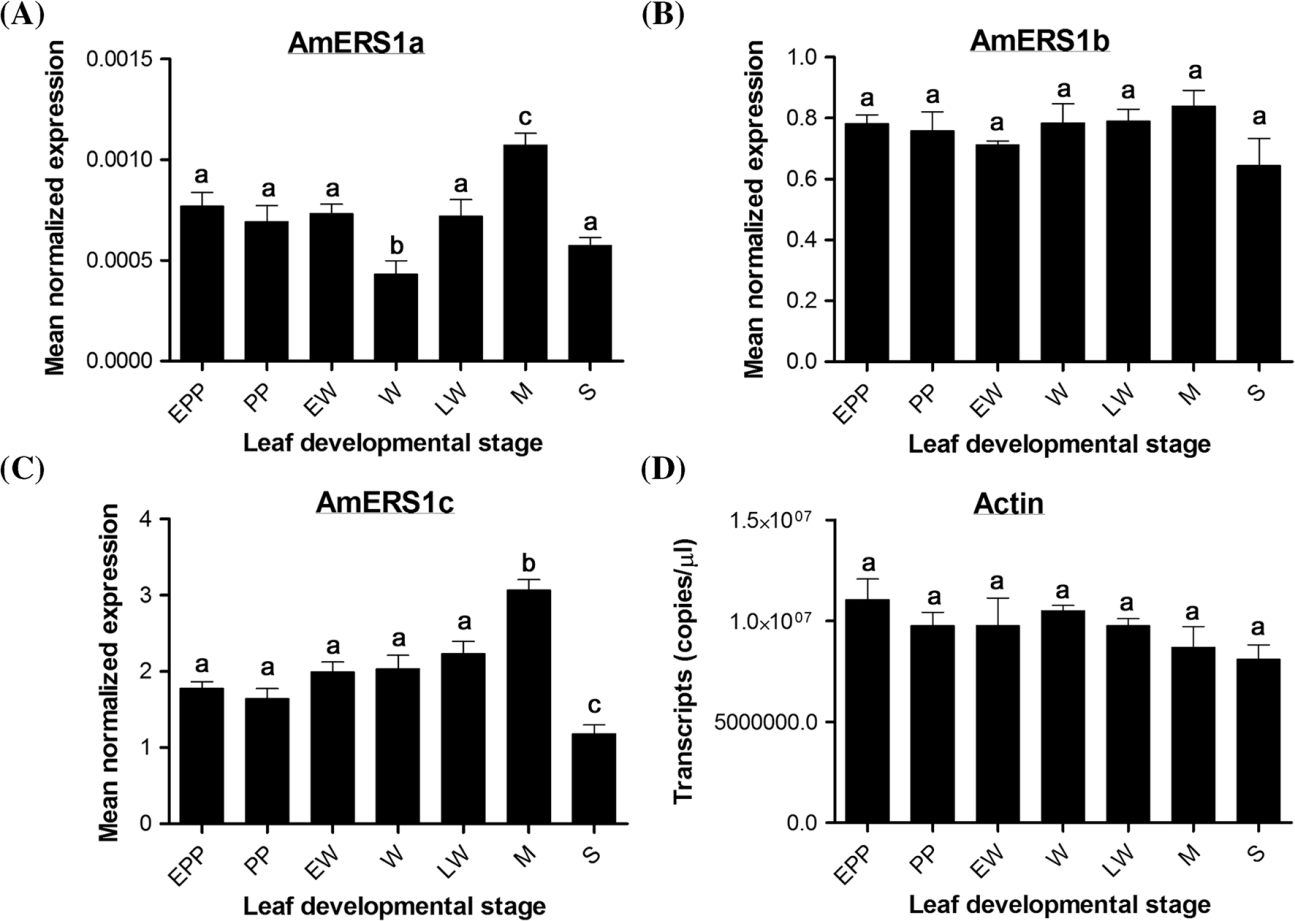
Normalized ethylene receptor transcript levels at different stages of leaf development. **a** Normalized AmERS1a transcript levels at different stages of leaf development. Window stage leaves, in which PCD is occurring, had significantly lower transcript levels AmERS1a than all other leaf developmental stages. Mature leaves, in which perforation formation is complete, had significantly higher AmERS1a transcript levels than all the other developmental stages. The transcript levels declined during leaf senescence. There was no significant difference in AmERS1b transcript levels throughout leaf development (**b**). AmERS1c had the highest transcript levels during the mature stage, while senescent leaves (in which PCD is occurring) had the lowest transcript levels (**c**). **d** Actin transcripts were constitutively expressed in all stages of lace plant leaf development. Bars represent SE (n ≥ 12). *Means with the same letters* are not significantly different (*P* > 0.05)

Quantitative PCR results showed that AmERS1a transcript levels are similar from early preperforation to early window stage (Fig. 6a). The AmERS1a transcript levels declined significantly (*P* > 0.05) during the window stage, in which perforation formation and PCD were occurring. During the mature stage, where PCD and perforation formation are no longer occurring, AmERS1a transcripts increased to the highest levels. The levels, however, declined significantly (*P* > 0.05) during leaf senescence. AmERS1b was constitutively expressed throughout leaf development (Fig. 6b). AsERS1c was constitutively expressed from early preperforation to late window stage (Fig. 6c). However, similar to AmERS1a, the AmERS1c transcript levels increased significantly (*P* > 0.05) during the mature stage. AmERSlc transcript levels also declined significantly to the lowest levels during leaf senescence. Of the three lace plant ethylene receptors, AmERS1c generally appeared to have the highest transcript levels in leaves, followed by AmERS1b, and AmERS1a had the least transcript levels throughout leaf development. Actin, the reference gene, was constitutively expressed throughout leaf development (Fig. 6d).

### Expression levels of AmERS1a, AmERS1b and AmERS1c in PCD and NPCD cells

**Fig. 7:**
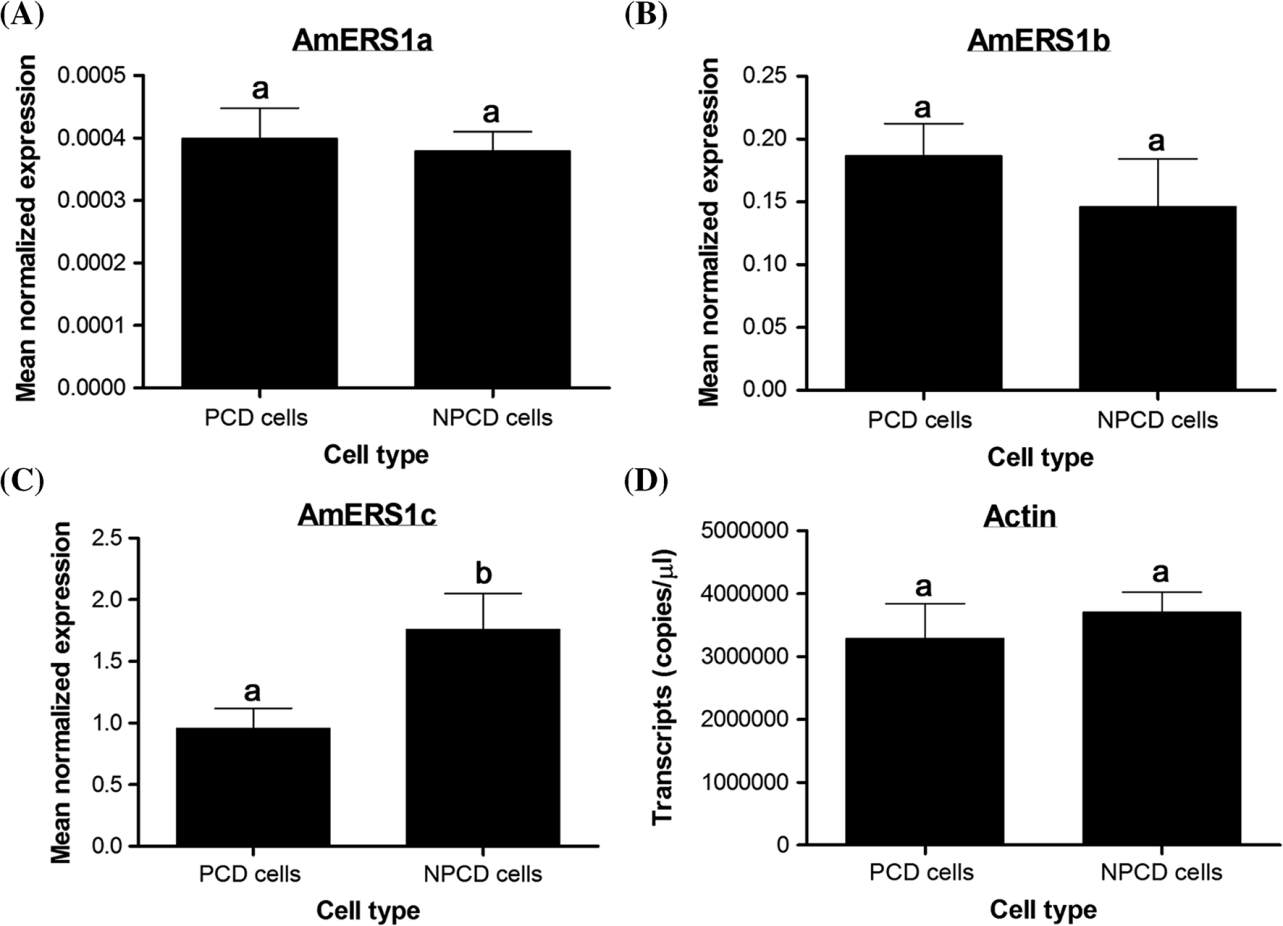
Ethylene receptor levels between PCD and NPCD cells. AmERS1a and AmERS1b did not have significant difference in transcript expression between PCD and NPCD cells (**a** and **b** respectively). Transcript levels for AmERS1c, the most abundant ethylene receptor, were significantly higher in NPCD than in PCD cells (**c**). **d** Reference gene, actin, did not show significant difference in transcription expression between the two types of cells. *Bars* represent SE (n ≥ 12). *Means with the same letters* are not significantly different (*P* > 0.05)

To further investigate the role of ethylene receptors in lace plant perforation formation and PCD, transcript levels between the dying (PCD) and non-dying (NPCD) cells were determined (Fig. 7). The cells were separated and isolated from window stage leaves using a Zeiss PALM Laser Capture Microdissection and Imaging System. AmERS1a and AmERS1b transcript levels were not significantly different between PCD and NPCD cells (Fig. 7a, b). AmERS1c had significantly higher (*P* > 0.05) transcript levels in NPCD cells than in PCD cells (almost twofold; Fig. 7c). Even at the cellular level, AmERS1c had the highest transcript levels, then AmERS1b and lastly, AmERS1a. Actin was constitutively expressed between the two cell types (Fig. 7d).

## Discussion

Recent research shows ethylene is involved in regulation of PCD in lace plant during perforation formation and senescence, in a climacteric-like pattern (Dauphinee et al. 2012). They showed that ethylene production peaks during the window and senescence stages, both in-which PCD is occurring. Lace plant is a unique example of ethylene climacteric-like behaviour during leaf morphogenesis through PCD. To determine the role of ethylene perception in regulation of lace plant leaf development and PCD, through ethylene receptors, we isolated three lace plant receptors. These ethylene receptors, AmERS1a, AmERS1b and AmERS1c, showed high sequence similarity to other monocot ethylene receptors, from maize and rice. Rice has five ethylene receptors and they have been divided into two subfamilies (Bleecker 1999; Yau et al. 2004). Maize consists of four ethylene receptors and they also divided into the same two categories found in rice. The three lace plant ethylene receptors share more characteristics with the monocot subfamily I (OsERS1, OsERS2, ZmERS1a and ZmERS1b) than subfamily II (OsETR2, OsETR3, OsETR4, ZmETR2a and ZmETR2b) receptors. The subfamily I (or ERS) monocot and lace plant receptors all have the conserved residues within the histidine kinase domain and lack a receiver domain. Conversely, subfamily I (or ETR) receptors in maize and rice lack all or some of the essential residues within their histidine kinase domain an posses a receiver domain. Phylogenetic analysis, based on amino acid sequence similarity, also show that the lace plant ethylene receptors are more similar to ERS than ETR monocot ethylene receptors. All three isolated lace plant receptors also seem to be ERS1 isoforms. This is also supported by the phylogenetic analysis, which grouped them with ZmERS1a, ZmERS1b and OsERS1.

It is most likely that the lace plant genome possesses subfamily II ethylene receptors as well. So far, all the plant species that have their ethylene receptors isolated have both subfamily I and II ethylene receptors. These include *Arabidopsis* (Bleecker et al. 1998), tomato (Klee and Tieman 2002), maize (Chen and Gallie 2010) and rice (Yau et al. 2004). Subfamily 1 ethylene receptors in *Arabidopsis* play a predominant role in regulation of ethylene responses (Wang et al. 2003; Shakeel et al. 2013). The ethylene receptors overlap in terms of functions during the control of ethylene responses. However, Wang et al. (2006) showed that the lack of a subfamily I receptor in *Arabidopsis* results in a constitutive ethylene response, in which the inhibitory effect of ethylene receptors in ethylene induced responses is lacking. Hall and Bleecker (2003) also showed that *Arabidopsis* subfamily 1 (*ers1* and *etr1*) double loss of function mutants are severely developmentally defective, providing more evidence for the paramount importance of subfamily I receptors in development and regulation of ethylene induced responses.

Ethylene receptors also have non-overlapping roles; some are mostly involved in pathogen responses (Knoester et al. 1998; Plett et al. 2009a), response to silver ions (McDaniel and Binder 2012), growth recovery after exposure to exogenous ethylene (Kim et al. 2011), trichome development (Plett et al. 2009a, b), and nutational bending (Binder et al. 2006; Kim et al. 2011). In the lace plant, ethylene receptors seem to play a role in leaf development during perforation formation through PCD. An ethylene receptor inhibitor, silver nitrate (AgNO_3_), reduced the number of perforations (Gunawardena et al. 2006). To determine the role of three lace plant ethylene receptors in leaf development and developmentally regulated PCD, we studied the transcript levels of each of the receptors throughout seven stages of lace plant leaf development. In general, AmERS1c had the highest transcript levels in leaf tissue. Its transcript levels were approximately 4000-fold the amount of AmERS1a and threefold the amount of AmERS1b. This suggests that AmERS1c plays a predominant role in ethylene perception during leaf development. AmERS1a also seems to be involved in lace plant PCD, despite its generally low transcript levels in leaves. Its transcript levels were significantly lower in window stage leaves, in which perforation formation and PCD occur. This is also when ethylene levels peak (Dauphinee et al. 2012). The AmERS1a transcript levels then significantly increase during the mature stage, when the perforation is complete and ethylene levels are low. During senescence, when PCD is occurring and ethylene levels peak again, AmERS1a levels are reduced. AmERS1c levels are significantly high in mature stage leaves and significantly low during senescence. In window stage leaves, AmERS1c levels are lower in PCD cells than in NPCD cells. Even though AmERS1a levels are generally significantly lower in the window stage leaves, its transcript levels are not significantly different between PCD and NPCD cells. AmERS1b is constitutively expressed throughout leaf development and between the two types of cells and therefore unlikely to play a significant role in regulation of PCD during perforation formation. AmERS1a and AmERS1c seem to be the key players in regulation of ethylene perception and regulation of ethylene-dependent PCD during perforation formation in lace plant.

**Fig. 8:**
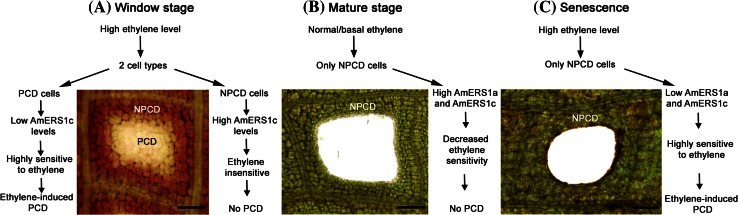
Proposed ethylene receptor expression pattern model during lace plant leaf development. **a** Illustration of the proposed AmERS1a and AmERS1c expression pattern model in window stage lace plant leaves. Non-PCD cells (NPCD) are outside the perforation site while PCD cells are within the perforation site. The *diagram* illustrates how each cell type responds to high ethylene levels in window stage leaves during PCD. **b** Illustration of the proposed ethylene receptor expression pattern model in mature stage lace plant leaves. At this stage ethylene levels are normal and no cells are undergoing PCD. The only cells remaining at this stage are NPCD cells and they have high AmERS1a and AmERS1c transcript levels. **c** During the senescence stage, when the entire leaf tissue dies, there is a peak in ethylene production. AmERS1a and AmERS1c levels are significantly low during this ethylene-induced PCD process. *Scale bars* 375 μm in A, and 150 μm in B and C

Ethylene receptors are negative regulators to the ethylene signal transduction pathway (Hall et al. 2007). The effect of ethylene on ethylene-induced responses is dependent on the amount of ethylene and ethylene receptors. Ethylene levels are known to vary between species, different developmental stages, and different tissues within a plant (Ievinsh and Ozola 1998). Also, plants are known to increase sensitivity to ethylene by either reducing their ethylene receptor levels or producing more endogenous ethylene (Chang et al. 1993; Zhao and Schaller 2004; reviewed in Arora 2005). A proposed model of how ethylene receptor (AmERS1a and AmERS1c) transcript and endogenous ethylene levels regulate perforation formation and PCD in the lace plant is illustrated in Fig. 8. In the lace plant, it has been shown in window stage leaves there are significantly higher ethylene levels, than in mature stage leaves (Dauphinee et al. 2012). Through ethylene biosynthesis inhibitor studies, it was shown that the high ethylene is necessary for perforation formation and PCD to occur (Dauphinee et al. 2012). In this high ethylene environment in window stage leaves, only PCD cells undergo PCD, and NPCD cells seem to be resistant to the high ethylene levels. This resistance can be attributed to the increase in AmERS1c transcript levels within NPCD cells that we observed in this study (Fig. 7). We hypothesize that since the PCD cells seem to lower their AMERS1c (the most abundant receptor by far) levels, they become susceptible to ethylene and the ethylene-induced PCD occurs in these cells. After being exposed to the high ethylene levels during the window stage, NPCD cells seem to maintain their high AmERS1c and increase their AmERS1a transcript levels to withstand ethylene induced PCD. These high transcript levels are evident in mature stage leaves where developmental PCD is no longer occurring as perforation formation is complete. Less ethylene is also produced in the mature leaves (Dauphinee et al. 2012). During senescence ethylene levels peak again (Dauphinee et al. 2012), and AmERS1a and AmERS1c transcript levels significantly decline, making the cells susceptible to ethylene and giving rise to the ethylene-regulated PCD in all the cells.

## Conclusions

The lace plant is an excellent model for studying cell biological aspects of PCD. It had been shown previously that the plant hormone ethylene plays an important role in regulation of lace plant PCD. Genetic regulation of developmentally regulated PCD in the lace plant has been unclear. This study provides some insight into how it may be genetically determined which cells are supposed to undergo PCD during perforation formation in the lace plant. The proposed model involving ethylene and ethylene receptors (Fig. 8) explains why despite being within the same leaf tissue and environment, some cells die and others survive. Ethylene has been implicated as the trigger and regulator in other plant PCD systems, but in the lace plant the intrinsic signal that triggers increases in endogenous ethylene production and adjustment of ethylene receptors to determine cell fate is still unknown. Three lace plant ethylene receptors were isolated in this study, all of them are subfamily I receptors. It is unlikely that more of the subfamily I receptors exist, but it is almost certain that lace plant has undiscovered subfamily II receptors. Isolating the remaining ethylene receptor family members and studying their expression patterns would provide more insight into how each of the receptors is involved in perforation formation in lace plant. Other genes within the ethylene biosynthesis and signal transduction pathways also need to be isolated and this will allow for more in-depth studies of the role of ethylene during perforation formation. Transcript level studies may also be supplemented with ethylene receptor mutants, to provide more insight into receptor function. Other genes that play a role in signalling, regulation and execution of lace plant PCD also need to be isolated and their roles investigated.
